# Loeffler endocarditis complicated by cardiogenic shock and angioinvasive *Fusarium fujikuroi* infection: a case report

**DOI:** 10.1093/ehjcr/ytag670

**Published:** 2026-09-12

**Authors:** Jack Simmons, Ahmed K Saleh, Preston Nicely, William Morgan, Stephen Clarkson

**Affiliations:** Tinsley Harrison Internal Medicine Residency Program, University of Alabama, 1720 2nd Ave S, Birmingham, AL 35294, USA; Division of Cardiovascular Diseases, University of Alabama, 1720 2nd Ave S, Birmingham, AL 35294, USA; Tinsley Harrison Internal Medicine Residency Program, University of Alabama, 1720 2nd Ave S, Birmingham, AL 35294, USA; Division of Cardiovascular Diseases, University of Alabama, 1720 2nd Ave S, Birmingham, AL 35294, USA; Division of Cardiovascular Diseases, University of Alabama, 1720 2nd Ave S, Birmingham, AL 35294, USA

**Keywords:** Loeffler endocarditis, Immunosuppression, Cardiogenic shock, Restrictive cardiomyopathy, Cardiac magnetic resonance imaging, Transthoracic echocardiogram, Case report

## Abstract

**Background:**

Loeffler endocarditis is a form of restrictive cardiomyopathy that affects up to half of patients with hypereosinophilic syndromes. Patients undergoing treatment of Loeffler endocarditis with immunosuppression are at increased risk of mixed cardiogenic and septic shock due to its restrictive ventricular physiology and treatment with immunosuppressive therapy.

**Case summary:**

A 64-year-old female with peripheral eosinophilia and heart failure with mildly reduced ejection fraction reported 6 months of fatigue, worsening lower extremity oedema, palpitations, dyspnoea on exertion, and orthopnoea. She was diagnosed with Loeffler endocarditis based on cardiac magnetic resonance imaging and was initiated on high-dose prednisone and rituximab. While undergoing treatment of a subsequent angioinvasive fungal infection, she developed mixed cardiogenic and septic shock requiring inotropic support and broad-spectrum antimicrobials. She was eventually weaned off inotropic support and was continued on long-term posaconazole for the angioinvasive fungal infection.

**Discussion:**

Patients undergoing treatment of Loeffler endocarditis with immunosuppression are at an increased risk of mixed cardiogenic and septic shock. Late-stage Loeffler endocarditis predisposes to cardiogenic shock due to restrictive ventricular physiology and thrombi obliterating the ventricular cavities, while immunosuppressive therapy increases risk of opportunistic infections. If immunosuppression is initiated before ventricular fibrosis occurs, progression of Loeffler endocarditis can be halted or reversed.

Learning points

- Immunosuppression increases risk of mixed septic and cardiogenic shock in Loeffler endocarditis complicated by restrictive ventricular physiology.
- Rapidly progressive necrotic skin lesions in immunosuppressed patients should raise suspicion for angioinvasive *Fusarium* spp. infection.
- If immunosuppression is initiated before irreversible fibrosis occurs, progression of inflammation, as evidenced by late gadolinium enhancement, can be prevented.

## Introduction

Loeffler endocarditis (LE) is an uncommon restrictive cardiomyopathy (RCM) that can present in up to 50% of patients with hypereosinophilic syndromes (HES).^1^ Treatment of LE includes immunosuppressive therapy, which can predispose to opportunistic infections. Patients with LE are at increased risk of cardiogenic shock, as the proinflammatory, hypercoagulable conditions associated with LE can lead to thrombi causing ventricular filling defects and restrictive ventricular physiology.^1,2^ These factors place patients undergoing treatment of LE at an increased risk of mixed cardiogenic and septic shock.

## Summary figure

**Figure:**
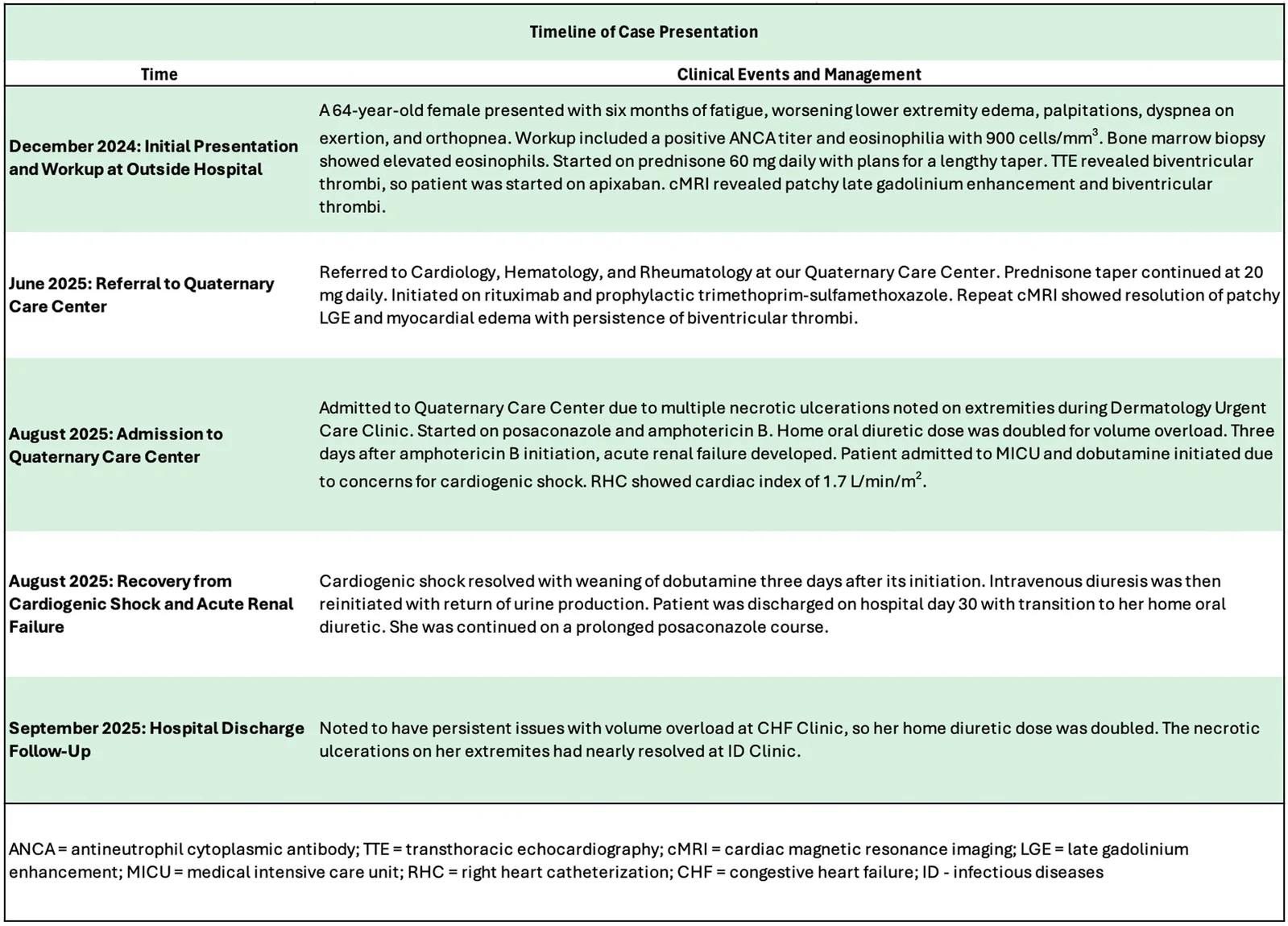


## Case presentation

A 64-year-old female with suspected eosinophilic granulomatosis with polyangiitis (EGPA) presented to our hospital with 6 months of fatigue, worsening lower extremity oedema, dyspnoea on exertion, orthopnoea, photophobia, blurry vision, and bilateral wrist pain. Vitals disclosed a temperature of 37.9°C, blood pressure of 100/70 mmHg, heart rate of 92 beats per minute, and oxygen saturation of 100% on room air. Physical exam revealed 3+ pitting oedema up to the knees bilaterally, no jugular venous distention, regular rate and rhythm, and diffuse necrotic ulcerations on all extremities.

## Initial presentation

Work-up at an outside hospital 6 months prior to presentation revealed eosinophilia with 900 cells/mm^3^ and positive anti-neutrophil cytoplasmic antibody (ANCA). Bone marrow biopsy showed an elevated eosinophil count but no myeloid neoplasm. Transthoracic echocardiography (TTE) revealed an ejection fraction (EF) of 45%–50% and biventricular apical thrombi. Cardiac magnetic resonance imaging (cMRI) showed lateral and inferior wall mid-myocardial oedema, diffuse patchy late gadolinium enhancement (LGE), and biventricular apical thrombi (*Figure 1A, 1B*). Left heart catheterization revealed no coronary artery disease. The differential diagnosis included LE, drug-induced eosinophilic myocarditis, cardiac sarcoidosis, amyloidosis, Danon disease, and Anderson–Fabry disease. Due to peripheral eosinophilia, positive ANCA antibody titre, and ophthalmologic and musculoskeletal involvement, LE secondary to EGPA was the leading diagnosis. Although she did not meet 2022 EULAR diagnostic criteria for EGPA, she received empiric immunosuppression due to presumed EGPA. She did not receive any common causes of drug-induced eosinophilic myocarditis, lowering its suspicion. Endomyocardial biopsy was not pursued due to the risk of thrombus embolization. Apixaban was initiated for its ease of monitoring over warfarin. Rheumatology initiated prednisone 60 mg daily for suspected EGPA. Ophthalmology recommended an extended prednisone taper for a suspected left corneal ulcer. During the following months, she had multiple hospitalizations for hypervolemia requiring diuresis.

**Figure 1:**
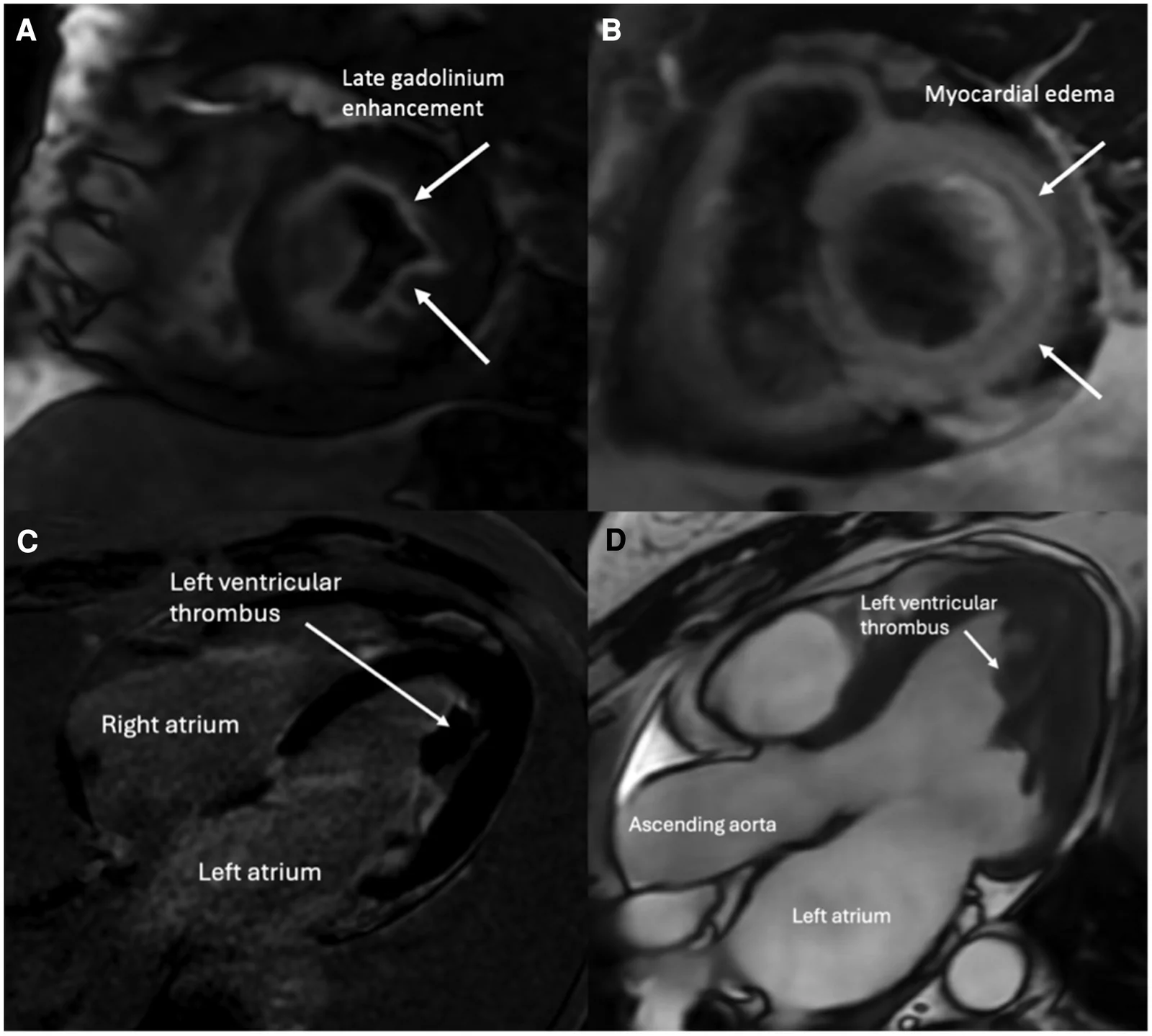
*A*) Cardiac magnetic resonance short-axis view demonstrating subendocardial late gadolinium enhancement in the anterior, lateral, and inferior walls of the left ventricle. Diffuse subendocardial late gadolinium enhancement is common in Loeffler endocarditis cardiac magnetic resonance imaging. *B*) T2-weighted cardiac magnetic resonance short-axis view demonstrating mid-myocardial oedema most appreciable in the lateral and inferior walls of the left ventricle. Myocardial oedema is not specific for Loeffler endocarditis, but can indicate active inflammation. *C*) Cardiac magnetic resonance axial view demonstrating a thrombus attached to the left ventricular wall. Thrombi are consistent with the second, or thrombotic, stage of Loeffler endocarditis. *D*) Cardiac magnetic resonance axial view showing a thrombus attached to the left ventricular wall.

## Pre-hospital course

After referral to our hospital, she received a TTE which showed eccentric left ventricular (LV) hypertrophy, normal left and right ventricular EF, large LV thrombus and possible right ventricular thrombus, septal e’ 4.1 cm/s, lateral e’ 6.1 cm/s, septal E/e’ ratio 17.1, left atrial volume index 41.1 mL/m^2^, grade II diastolic dysfunction, mild–moderate tricuspid regurgitation, and right ventricular systolic pressure of 42.3 mmHg (*Figure 2A, 2B*). Repeat cMRI showed resolution of patchy LGE, no myocardial fibrosis, left and right ventricular EF of 50% and 39%, respectively, a 24 × 13 mm LV thrombus, and an 8 mm right ventricular apical thrombus (*Figure 1C, 1D*). By the time of referral, her prednisone had been tapered down to 30 mg daily. Rituximab was initiated due to concern for systemic involvement of EGPA. Three weeks after initiating rituximab, the patient developed multiple rapidly progressive necrotic ulcerations on her extremities; lesion biopsies returned positive for angioinvasive *Fusarium fujikuroi* (*Figure 3A, 3B*), so she was admitted to our hospital.

**Figure 2:**
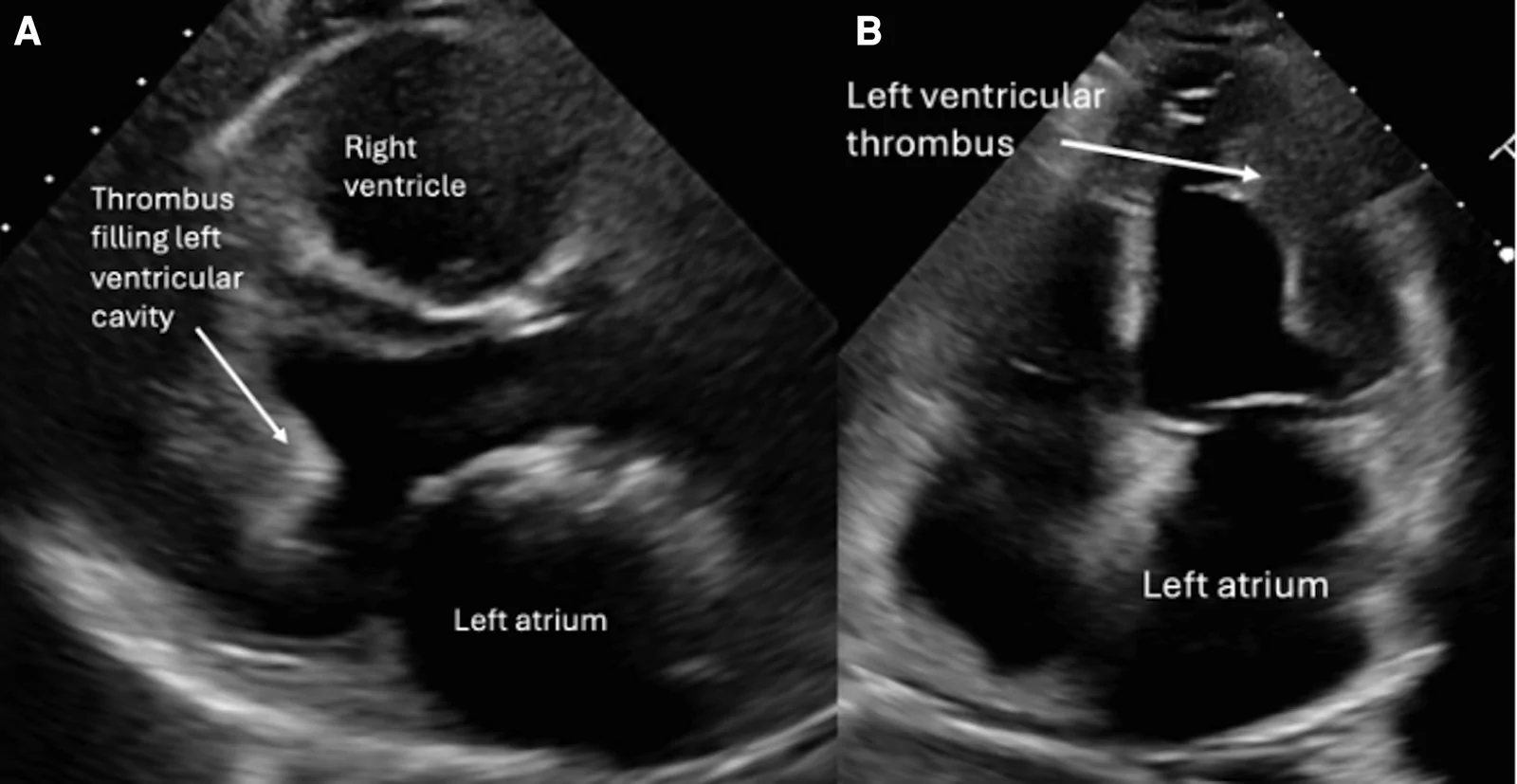
*A*) Parasternal long axis view showing thrombus intruding into the left ventricular cavity, consistent with the second, or thrombotic, stage of Loeffler endocarditis. *B*) Apical four-chamber view during early systole showing infiltration of thrombus into left ventricular cavity.

**Figure 3:**
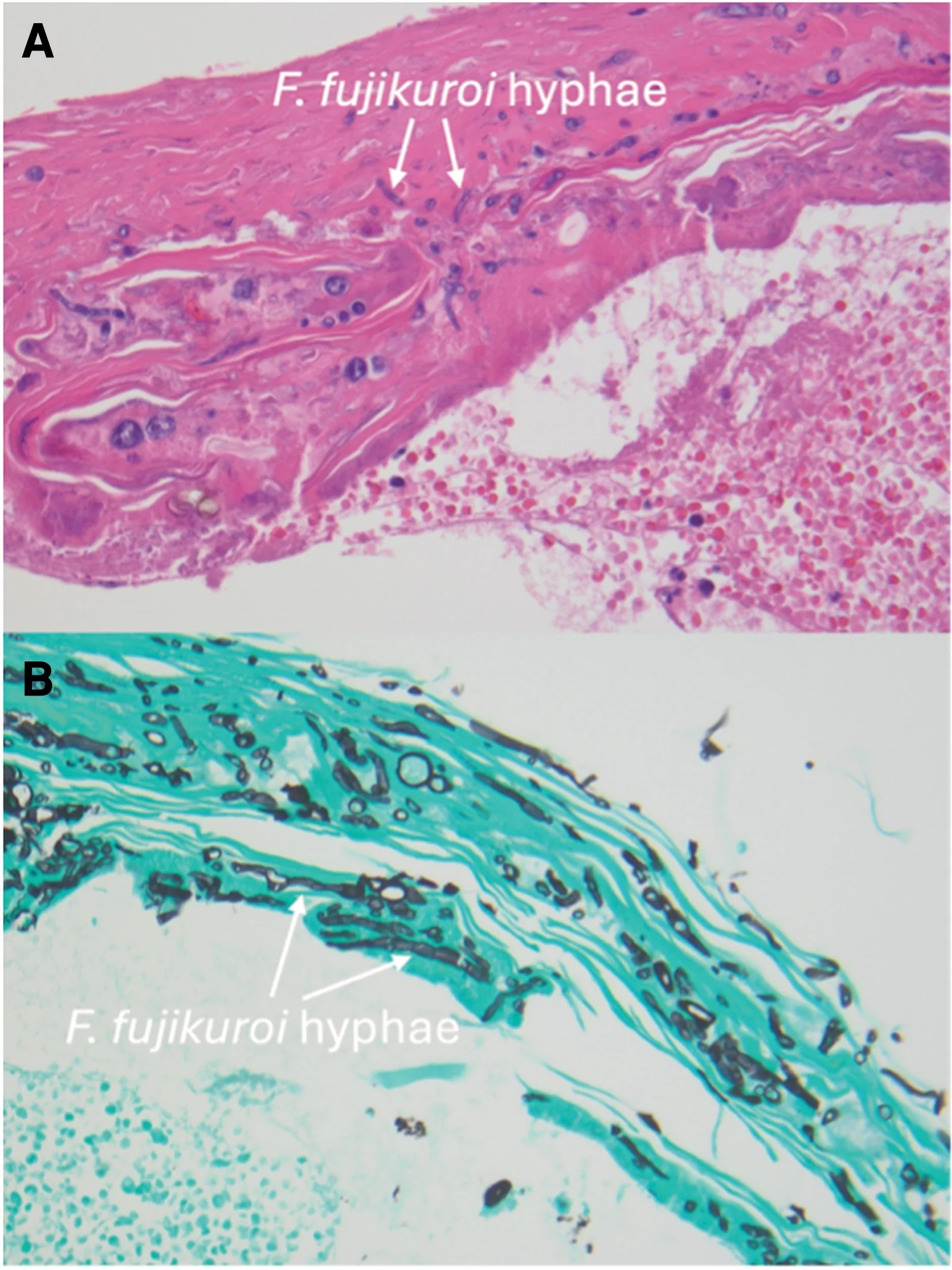
*A*) Haematoxylin and eosin stain showing *Fusarium fujikuroi* hyaline septated hyphae within epidermis. Image *B*) Grocott methenamine silver stain revealing *F. fujikuroi* septated hyphae within epidermis.

## Hospital course

Upon admission, white blood cell count was 25.2 × 10^3^ cells/mm^3^ and blood cultures were negative. Posaconazole and amphotericin B were started for the angioinvasive *Fusarium* infection. Torsemide 120 mg twice daily was initiated for significant hypervolemia. Three days after amphotericin B initiation, she developed acute renal failure and was admitted to the Medical Intensive Care Unit (MICU) for continuous renal replacement therapy (CRRT). Repeat TTE showed LVEF 60%–65% and LV outflow tract velocity time integral 12.0 cm. Right heart catheterization revealed a cardiac index (CI) of 1.7 L/min/m^2^, right atrial pressure of 21 mmHg, pulmonary capillary wedge pressure of 30 mmHg, mean pulmonary arterial pressure of 30 mmHg, pulmonary vascular resistance of 240 dynes/s/cm^5^, and systemic vascular resistance (SVR) of 1935 dynes/s/cm^5^. An elevated SVR, a depressed CI, and mixed venous oxygen saturation of 35% suggested a primarily cardiogenic rather than septic component driving her shock, while normal LVEF suggested a mostly diastolic component to her cardiogenic shock. Dobutamine 5 mcg/kg/min was initiated for inotropic support; milrinone was avoided due to her concurrent renal failure. Three days later, she was weaned off dobutamine, and CRRT was weaned the next day. After 11 days in the MICU, she was transferred to the floor. Blood cultures were obtained due to worsening foot wounds and grew vancomycin-resistant *Enterococcus faecium*, for which she received seven days of daptomycin. She was transitioned to her home oral diuretic and metoprolol before discharge.

At 1-month advanced heart failure follow-up, her home furosemide was increased to 40 mg twice daily due to issues with volume control. At 3-month follow-up, she noted stable oedema, and spironolactone 25 mg was added. She was not a transplant candidate due to improved functional status and stable NYHA Class III symptoms. Rheumatology discontinued her prednisone taper 3 months after discharge due to symptomatic improvement. She received 6 months of posaconazole for the angioinvasive fungal infection.

## Discussion

The combination of the restrictive physiology of LE and immunosuppressive treatment can predispose patients to mixed cardiogenic and septic shock. RCM, such as LE, require adequate preload due to elevated diastolic filling pressures and small ventricular cavities.^3^ Caution must be taken with the use of inotropic medications, as arrhythmias are more prevalent in RCM.^3^ Use of mechanical circulatory support in LE is extremely rare and must be chosen with care due to the typically small ventricular cavities.^2,4,5^

The initial, or acute necrotic phase, of LE is defined by endomyocardial necrosis and apoptosis triggered by eosinophilia and can cause endomyocardial thickening. The second, or thrombotic phase, can lead to intracardiac thrombi, typically in the ventricular apices, stroke, pulmonary embolism, and rarely sudden cardiac death. Vegetations may arise from the ventricular walls with possible extension to valvular leaflets.^6^ Thrombi can obstruct the ventricular cavities, causing filling defects.^1,6^ The third and final stage is the fibrotic stage, which exhibits RCM physiology and is driven by fibro-inflammatory processes and remodelling of granulation tissue.^1^ MRI classically shows subendocardial LGE in all three stages and helps identify tissue fibrosis and thrombus characteristics.

Corticosteroids are the first-line treatment for LE. Alternative therapies include hydroxyurea, azathioprine, and tyrosine kinase inhibitors; rituximab can be used if treating concomitant HES.^1^ High-dose, prolonged corticosteroid regimens predispose patients to infection; additional therapies, such as rituximab, only further increase the risk. Early initiation of immunosuppressive therapy is imperative to reduce mortality and prevent progression to the fibrotic stage, where myocardial fibrosis becomes irreversible.^7^ Our patient was started on immunosuppressives in the thrombotic phase before ventricular fibrosis occurred, with resolution of LGE noted on repeat cMRI. If present, thrombi can be treated with direct oral anticoagulants instead of vitamin K antagonists when frequent international normalized ratio checks are impractical or if therapeutic levels are difficult to achieve.^8^

Management of *Fusarium* infections is challenging due to frequent multidrug resistance and their predilection for immunocompromised hosts. Amphotericin B, posaconazole, and voriconazole are the most effective antifungal agents, and combination therapy may be warranted in severely immunocompromised patients.^9^

## Lead author biography


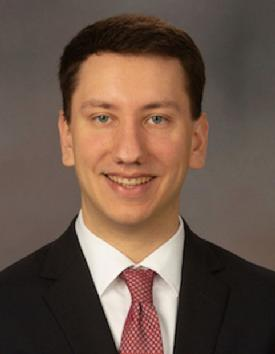
Dr. Jack Simmons is an Internal Medicine Resident at the University of Alabama at Birmingham. He has a strong clinical interest in cardiology, particularly multimodal imaging, preventive cardiology, and cardiogenomics.

## Data Availability

The data underlying this article will be shared on reasonable request to the corresponding author.

## Notes

**Consent:** The authors confirm that written consent for submission and publication of this case report, including images and associated text, has been obtained from the patient, in line with COPE guidance.
